# The Impact of Mock Code Simulation on the Resuscitation Practice and Patient Outcome for Children With Cardiopulmonary Arrest

**DOI:** 10.7759/cureus.9197

**Published:** 2020-07-15

**Authors:** Tarek R Hazwani, Arwa Alosaimi, Manal Almutairi, Naila Shaheen, Zahra Al Hassan, Mohannad Antar

**Affiliations:** 1 Pediatric Intensive Care, King Abdulaziz Medical City, Ministry of National Guard - Health Affairs, Riyadh, SAU; 2 Pediatric Critical Care, College of Medicine, King Saud Bin Abdulaziz University for Health Sciences, Riyadh, SAU; 3 Pediatrics, College of Medicine, King Saud Bin Abdulaziz University for Health Sciences, Riyadh, SAU; 4 Family Medicine, College of Medicine, King Saud Bin Abdulaziz University for Health Sciences, Riyadh, SAU; 5 Biostatistics and Bioinformatics, King Abdullah International Medical Research Center, Riyadh, SAU; 6 Nursing, Ministry of National Guard - Health Affairs, Riyadh, SAU; 7 Pediatrics, Ministry of National Guard - Health Affairs, Riyadh, SAU

**Keywords:** simulation, mock code, resuscitation, outcome, cardiac arrest, team performance

## Abstract

Background

Cardiopulmonary arrest is an uncommon event in pediatric patients. Additionally, physicians-in-training see far fewer cardiopulmonary arrest events. Therefore, they have limited confidence in their resuscitation skills. Mock code training with active participation and debriefing may be an effective tool to fill this gap in experience. The aims of the study were to assess the impact of a mock code simulation program on patient outcome for children with cardiopulmonary arrest in a tertiary pediatric academic center and provide evidence that code simulations can improve the quality of cardiopulmonary resuscitation (CPR).

Methods

This was a retrospective cohort study conducted in a tertiary academic center. This study had two phases: Phase 1 before the mock code simulation program began (pre-intervention) and Phase 2 after the mock code program began (post-intervention). The data were collected from pediatric patients with cardiopulmonary arrest during the study period who met the inclusion criteria, and variables included the survival rate at hospital discharge, CPR initiation time, time to the first dose of epinephrine, and the adherence rate to American Heart Association (AHA) guidelines.

Results

A total of 13 patients in the pre-intervention period and 19 patients in the post-intervention period were included. The results showed a significant improvement in team performance represented by a decrease in CPR initiation time post-intervention and improvement in AHA adherence; however, the results did not show a significant difference in the survival rate or mortality within 28 days of the cardiopulmonary arrest event between the pre- and post-intervention groups.

Conclusions

Mock code simulation was a helpful tool to enhance team performance and improve the quality of cardiac resuscitation and cardiac arrest recognition, while its impact on the survival rate was not significant in our study.

## Introduction

Cardiopulmonary arrest is considered to be an uncommon event in pediatric patients and requires timely and high-quality cardiopulmonary resuscitation (CPR) to improve outcomes [1,2]. Physicians-in-training see far fewer code-blue events than their predecessors did, so they have limited confidence in resuscitation skills [3]. Mock code simulation training with active participation and debriefing may be an effective educational tool to fill this gap in their experience [4]. Due to limited exposure to this situation, health care providers need to maintain and develop their resuscitation skills by going through these critical situations to prepare them to deal confidently with a future event [5,6]. Several studies have shown a reduction in pediatric CPR knowledge and skills within a short period of time after training if the skills are not maintained and practiced [6-8].

A mock code, which is defined as a simulation exercise with a mannequin/human patient simulator that has no respiratory effort and/or no carotid pulse, helps to encourage interprofessional teams to collaborate and improve their teamwork and fast response skills [9,10]. These capabilities are crucial to reach the best outcome when performing CPR in a real environment, as well as in the mock code scenario where patient safety is not a concern and the exercise allows us to measure and assess the performance level. This exercise can fill knowledge and practice gaps and prepare practitioners for real situations [11-13].

Moreover, conducting good debriefing after the simulation sessions and sustaining the program over time are other important elements that help to ensure a successful program and achieve better patient outcomes [6,8,14-17].

There are various approaches to resuscitation training. In situ simulation training holds the training session in a realistic environment and offers the opportunity to identify issues with the existing code processes, such as equipment availability, location, and functionality [12,18-20]. Several studies support the use of resuscitation training programs that are multidisciplinary sessions based on the latest resuscitation guidelines. Furthermore, mock code training programs minimize performance differences and help to improve the response time from the onset of loss of pulse to the initiation of chest compressions, the time spent administering the first dose of epinephrine, and defibrillation time [13,21-25].

Despite the educational model used in the mock code training program, the goal is to optimize the survival rate. Many of these programs found a strong correlation between the survival rate and high numbers of mock codes [12,26-28]. Therefore, it is suggested that the mock code simulation program be added to pediatric advance life support (PALS) training in pediatric residency programs to promote confidence in responding to pediatric resuscitation [7,8,15,16].

Few studies have assessed the impact of mock code simulations on pediatric patient survival. Overall, it appears that this kind of simulation-based training had a significant positive effect on performance in a cardiac arrest situation [6,8,12,13,15].

Our study aimed to assess the impact of a mock code simulation program on the resuscitation practice of resuscitation team, and patient outcome for children with cardiopulmonary arrest events in a tertiary pediatric academic center.

## Materials and methods

Simulation program design and participants

The mock code simulation program at King Abdullah Specialized Children Hospital, Riyadh, was established in 2015. This institution is the largest pediatric tertiary care medical center under the Ministry of National Guard - Health Affairs in Saudi Arabia. The hospital’s inpatient bed capacity is about 225, with the 25-bed pediatric intensive care unit (PICU) and nine specialized inpatient pediatric wards in which code simulation sessions have been conducted.

Pediatric Simulation Training and Education Program (PediSTEP) is a multidisciplinary simulation program and offers different types of simulation programs such as high-fidelity and low-fidelity simulation, interprofessional simulation, and in situ mock code simulation.

This in situ mock code simulation is held two to three times per month, at random time and place. It is announced as real code, allowing us to offer the training opportunity for team members in their real settings and work environment. The pediatric cardiac arrest team is composed of a pediatric resident as a team leader, pediatric ward nurses as first responders, pediatric intensive care nurse, respiratory therapist, pharmacist, and pediatric ward charge nurse.

Multiple scenarios have been designed, including different types of common arrhythmias with structured debriefing sessions that focus on CPR and resuscitation skills. A low-fidelity mannequin is used for this purpose, while other instruments used, such as defibrillators, oxygen, medications, suction machines, and resuscitation carts, are from actual ward settings.

Design, setting, and populations

This was a cohort retrospective study conducted at King Abdullah Specialized Children Hospital at King Abdulaziz Medical City in Riyadh, Saudi Arabia.

This study had two phases: Phase 1 before the mock code simulation program began (pre-intervention), between 1 June 2015 and 31 May 2016, and Phase 2 after the mock code program began (post-intervention), between 1 January 2017 and 31 December 2018; a six-month implementation period was excluded.

Ethics approval for the study was sought from our local Institutional Review Board (IRB) at King Abdullah International Medical Research Center prior to data collection, and as this was an observational retrospective study and no identifiers were collected, the IRB considered the study exempt from the need for informed consent.

All records of pediatric cardiopulmonary arrest occurring in the study periods have been reviewed. All patients aged one month to 14 years with cardiopulmonary arrest events that occurred in PICU or in the ER were excluded, as the settings of these two areas are different.

Outcomes and measurements

The primary aim of this study is to evaluate the impact of the mock code simulation program on team performance by assessing the CPR initiation time and time to the first dose of epinephrine.

The secondary aims were to evaluate the adherence of the code team to the American Heart Association (AHA) guidelines and to determine the impact of the mock code simulation program on the survival rate of pediatric patients with cardiopulmonary arrest at hospital discharge. AHA adherence has been reviewed through CPR records and CPR Committee reports.

Statistical analysis

Categorical variables collected were sex, admission location of cardiopulmonary arrest, specialty of the most responsible physician, the days when the CPR occurred (i.e., weekdays or weekends), timing of CPR (working hours/on-call hours), admission diagnosis, and comorbidities. The results are summarized as frequencies and corresponding p-values. Continuous variables age, weight, height, length of stay in the ward, time taken for the team leader to arrive, time to initiate CPR, duration of CPR, time taken to administer epinephrine, epinephrine doses, PICU stay duration, and length of stay in ward were compared between the pre- and post-intervention phases by using the Wilcoxon rank-sum test. The results are summarized as the means, standard deviations, and corresponding p-values. Odds ratios (ORs) were calculated for patient outcomes between pre- and post-intervention phases. The results are reported as ORs and corresponding 95% confidence intervals. AHA guidelines were compared between the pre- and post-intervention phases by using Fisher’s exact test. Significance was declared using a p-value less than 0.05. Analysis was carried out using SAS version 9.3 (SAS Institute Inc., Cary, NC).

## Results

The study sample was collected from the pre-mock code simulation phases. Between 1 June 2015 and 31 May 2016, 15 participants were excluded because they were not a real code or seizure. In the post-mock simulation program period (between 1 January 2017 and 31 December 2018) there were 23 participants, and four of them were excluded due to the same reasons. The pre- and post-intervention groups were similar overall (Table 1).

**Table 1:** Baseline characteristics of the pre- and post-intervention groups CPA, cardiopulmonary arrest. *p-value is based on the Wilcoxon rank-sum test. **p-value is based on the Fisher exact test.

Characteristics	Pre-intervention group (n=13)	Post-intervention group (n=19)	p-value
Age (months) (mean±SD)	12.61±16.07	39.80±41.50	0.043
Age (years) (mean±SD)	1.05±1.34	3.32±3.46	0.043
Height	64.73±22.17	73.71±26.74	0.459*
Weight	6.27±4.46	11.69±7.26	0.012*
Gender, n (%)			
Male	10 (76.92)	10 (52.63)	0.267**
Female	3 (23.08)	9 (47.37)	
Admission location of CPA, n (%)			
General pediatric wards	8 (61.54)	11 (57.89)	
High dependency unit	3 (23.08)	7 (36.84)	0.566**
Surgery wards	2 (15.38)	1 (5.26)	
Most responsible physician, n (%)			
General pediatric	9 (69.23)	15 (78.95)	
Surgery	3 (23.08)	3 (15.79)	0.830**
Other specialty	1 (7.69)	1 (5.26)	
CPA days, n (%)			
Weekdays	9 (69.23)	11 (57.89)	0.712**
Weekends	4 (30.77)	8 (42.11)	
CPA timings, n (%)			
Working hours	5 (38.46)	7 (36.84)	1.000**
On-call hours	8 (61.54)	12 (63.16)	
Admission diagnosis, n (%)			
Respiratory	8 (61.54)	10 (52.63)	
Cardiac	0	3 (15.79)	
Sepsis	0	2 (10.53)	0.485**
Gastrointestinal	1 (7.69)	1 (5.26)	
Oncologic	1(7.69)	0	
Neurologic	3 (23.08)	3 (15.79)	
Others			
Comorbidities, n (%)			
Neurological (yes)	6 (46.15)	7 (3.84)	0.720**
Cardiac (yes)	7 (53.85)	5 (26.32)	0.150**
Pulmonary (yes)	3 (23.08)	5 (27.78)	1.000**
Immunology (yes)	3 (23.08)	7 (36.84)	0.467**
Gastroenterology (yes)	3 (23.08)	2 (10.53)	0.374**
Post-surgery (yes)	1 (7.69)	0	0.406**

The results showed a significant decrease in CPR initiation time by 22 seconds (p=0.031); however, no significant changes were noticed in the length of stay either in the hospital or in the PICU (Table 2).

**Table 2:** Cardiopulmonary arrest-related characteristics for pre- and post-intervention periods CPR, cardiopulmonary resuscitation. p-values are based on the Wilcoxon rank-sum test.

Characteristics	Pre-intervention group (n=13)	Post-intervention group (n=19)	p-value
Length of stay in ward (days) (mean±SD)	9.69±15.32	13.32±22.90	0.448
Time taken for the team leader to arrive (minutes) (mean±SD)	2±0.82	1.68±1.11	0.334
Time to initiate CPR (minutes) (mean±SD)	1.38±0.51	1.16±0.69	0.031
CPR duration (minutes) (mean±SD)	22.54±22.83	10.0±14.50	0.022
Time taken to give the first dose of epinephrine (minutes) (mean±SD)	3.38±1.33	2.21±2.12	0.067
Epinephrine doses (mean±SD)	5.23±5.73	3.50±5.49	0.378

The results did not show a significant difference in the survival rate or mortality within 28 days of the cardiopulmonary arrest event between the pre- and post-intervention groups (Table 3).

**Table 3:** Patient outcome comparison between pre- and post-intervention groups CPA, cardiopulmonary arrest. p-values are based on the Fisher exact test.

Variables	Pre-intervention group (n=13)	Post-intervention group (n=19)	p-value
Patient outcome, n (%)			
Dead	5 (38.46)	4 (21.05)	0.426
Alive	8 (61.54)	15 (78.95)	
Mortality within 28 days of CPA, n (%)			
Yes	6 (50)	6 (31.58)	0.452
No	6 (50)	13 (68.42)	

These patient outcome results were similar to the data analysis using odds ratios between the pre- and post-intervention groups, which revealed that it was 2.3 times more likely to have surviving cases in the post-intervention phase than in the pre-intervention phase. However, the 95% confidence interval includes 1, which indicates that the results are not significant (Table 4).

**Table 4:** Patient outcome odds ratios between pre- and post-intervention groups PICU, pediatric intensive care unit; OR, odds ratio. The reference groups are alive cases.

Variables	OR	95% CI
Patient outcome (dead vs. alive)	2.343	0.487-11.265
PICU discharge status (dead vs. alive)	3.750	0.661-21.251

Regarding the AHA adherence, it was observed that adherence was better in some elements, such as CPR initiation time (p=0.019) and electrical therapy for shockable rhythm (p=0.007), while the results for other elements did not significantly change between the pre- and post-intervention phases (Table 5).

**Table 5:** Adherence to AHA guidelines pre- and post-intervention AHA, American Heart Association; BLS, basic life support; PTL, pediatric resident team leader; PEA, pulseless electrical activity; VT, ventrical tachycardia; VF, ventrical fibrillation; ASY, asystole.

AHA guidelines	Pre-intervention group (n=13)			Post-intervention group (n=19)			p-value
	Met	Not met	Not applicable	Met	Not met	Not applicable	
BLS initiated in less than 1 min from arrest time	9 (69.23)	4 (30.77)	0	19 (100)	0	0	0.019
Epinephrine administered within 5 min of arrest	8 (61.54)	4 (30.77)	1 (7.69)	11 (57.89)	1 (5.26)	7 (36.84)	0.051
PEA/ASY or shock delivered if VT/VF	8 (61.54)	5 (38.46)	0	17 (89.47)	2 (10.53)	0	0.090
Every 2 min pulse time documented	13 (100)	0	0	19 (100)	0	0	-
Every 2 min identification of rhythm (after PTL arrival)	8 (61.54)	5 (38.46)	0	16 (84.21)	3 (15.79)	0	0.219
Every 2 min pulse checked	0	1 (7.69)	12 (92.31)	0	0	19 (100)	0.406
Every 2 min Joules selected in case of shockable rhythm	3 (23.08)	9 (69.23)	1 (7.69)	11 (57.89)	1 (5.26)	7 (36.84)	0.0007
Epinephrine documented for non-shockable rhythm (PEA and ASY) according to AHA guidelines	0	1 (7.69)	12 (92.31)	0	0	19 (100)	0.406
Epinephrine documented for shockable rhythm (VF and pulseless VT) according to AHA guidelines	0	1 (7.69)	12 (92.31)	0	0	19 (100)	0.406
Amiodarone given with the correct dose according to AHA guidelines	0	1 (7.69)	12 (92.31)	0	0	19 (100)	0.406
Amiodarone given at the correct time according to AHA guidelines	3 (23.08)	6 (46.15)	4 (30.77)	5 (26.32)	2 (10.53)	12 (63.16)	0.07

## Discussion

Overall, the results in this study showed a significant improvement in team performance represented by a decrease in CPR initiation time after the mock code simulation program, in both the CRP initiation time analysis and the AHA adherence analysis, as both revealed faster CPR initiation, which may reflect that cardiac arrest recognition improved after the mock code simulation program, and the first responders initiated chest compression earlier. Other studies reported the same results related to the CPR initiation time, which was part of the team dynamics improvement in some of them [21,25,29]. In our study, we did not find significant changes in the survival rate after the mock code simulation program, but the small sample size, which is one of the major study limitations, may have affected the results and made these changes difficult to trace, while the results of other studies showed better outcomes after implementing mock code simulation programs [30]. Other factors, such as comorbidities and the nature of the disease at admission, should also be considered and make drawing conclusion about the study outcomes more challenging, especially because cardiopulmonary arrest events are still considered rare events in pediatric patients.

## Conclusions

The mock code simulation program can enhance overall CPR performance by improving cardiac arrest recognition and CPR initiation time and increasing adherence to AHA guidelines. These results suggest advantages of the mock simulation program in terms of team performance, while its impact on survival rate is not clear in this small sample. Thus, future research with a larger study sample size might show a significant relation between the mock code simulation program and survival.

## References

[1] Topjian A, Berg R, Nadkarni V (2008). Pediatric cardiopulmonary resuscitation: advances in science, techniques, and outcomes. Pediatrics.

[2] Peberdy M, Kaye W, Ornato J (2003). Cardiopulmonary resuscitation of adults in the hospital: a report of 14 720 cardiac arrests from the National Registry of Cardiopulmonary Resuscitation. Resuscitation.

[3] Mickelsen S, McNeil R, Parikh P, Persoff J (2011). Reduced resident “code blue” experience in the era of quality improvement: new challenges in physician training. Acad Med.

[4] Van Schaik S, Von Kohorn I, O'Sullivan P (2008). Pediatric resident confidence in resuscitation skills relates to mock code experience. Clin Pediatr (Phila).

[5] Fiedor M (2004). Pediatric simulation: a valuable tool for pediatric medical education. Crit Care Med.

[6] Nadel F, Lavelle J, Fein J, Giardino A, Decker J, Durbin D (2000). Assessing pediatric senior residents’ training in resuscitation: fund of knowledge, technical skills, and perception of confidence. Pediatr Emerg Care.

[7] Hunt E (2006). Simulation of pediatric trauma stabilization in 35 North Carolina emergency departments: identification of targets for performance improvement. Pediatrics.

[8] Hunt E, Walker A, Shaffner D, Miller M, Pronovost P (2008). Simulation of in-hospital pediatric medical emergencies and cardiopulmonary arrests: highlighting the importance of the first 5 minutes. Pediatrics.

[9] Zideman D, De Buck E, Singletary E (2015). European Resuscitation Council guidelines for Resuscitation 2015 Section 9. First aid. Resuscitation.

[10] Szögedi I, Zrínyi M, Betlehem J, Újváriné A, Tóth H (2010). Training nurses for CPR: support for the problem-based approach. Eur J Cardiovasc Nurs.

[11] Tofil N, Lee White M, Manzella B, McGill D, Zinkan L (2009). Initiation of a pediatric mock code program at a children's hospital. Med Teach.

[12] Andreatta P, Saxton E, Thompson M, Annich G (2011). Simulation-based mock codes significantly correlate with improved pediatric patient cardiopulmonary arrest survival rates. Pediatr Crit Care Med.

[13] Knight L, Gabhart J, Earnest K, Leong K, Anglemyer A, Franzon D (2014). Improving code team performance and survival outcomes. Crit Care Med.

[14] Miller K, Riley W, Davis S, Hansen H (2008). In situ simulation: a method of experiential learning to promote safety and team behavior. J Perinat Neonatal Nurs.

[15] Van Schaik S, Plant J, Diane S, Tsang L, O'Sullivan P (2011). Interprofessional team training in pediatric resuscitation: a low-cost, in situ simulation program that enhances self-efficacy among participants. Clin Pediatr (Phila).

[16] Grant E, Marczinski C, Menon K (2007). Using pediatric advanced life support in pediatric residency training: does the curriculum need resuscitation?. Pediatr Crit Care Med.

[17] Curran V, Fleet L, Greene M (2012). An exploratory study of factors influencing resuscitation skills retention and performance among health providers. J Contin Educ Health Prof.

[18] Trainor J, Krug S (2000). The training of pediatric residents in the care of acutely ill and injured children. Arch Pediatr Adolesc Med.

[19] Herbers M, Heaser J (2016). Implementing an in situ mock code quality improvement program. Am J Crit Care.

[20] Hazwani T, Harder N, Shaheen N (2020). Effect of a pediatric mock code simulation program on resuscitation skills and team performance. Clin Simul Nurs.

[21] Huseman K (2012). Improving code blue response through the use of simulation. J Nurses Staff Dev.

[22] Wehbe-Janek H, Lenzmeier C, Ogden P (2012). Nurses’ perceptions of simulation-based interprofessional training program for rapid response and code blue events. J Nurs Care Qual.

[23] Kleinman M, Chameides L, Schexnayder S (2010). Part 14: pediatric advanced life support. 2010 American Heart Association guidelines for cardiopulmonary resuscitation and emergency cardiovascular care. Circulation.

[24] Kudenchuk P, Redshaw J, Stubbs B (2012). Impact of changes in resuscitation practice on survival and neurological outcome after out-of-hospital cardiac arrest resulting from nonshockable arrhythmias. Circulation.

[25] Ross J, Trainor J, Eppich W, Adler M (2013). Impact of simulation training on time to initiation of cardiopulmonary resuscitation for first-year pediatrics residents. J Grad Med Educ.

[26] Cappelle C, Paul R (1996). Educating residents: the effects of a mock code program. Resuscitation.

[27] Falck A, Escobedo M, Baillargeon J, Villard L, Gunkel J (2003). Proficiency of pediatric residents in performing neonatal endotracheal intubation. Pediatrics.

[28] Toback S, Fiedor M, Kilpela B, Reis E (2006). Impact of a pediatric primary care office-based mock code program on physician and staff confidence to perform life-saving skills. Pediatr Emerg Care.

[29] Delac K, Blazier D, Daniel L, N-Wilfong D (2013). Five Alive: using mock code simulation to improve responder performance during the first 5 minutes of a code. Crit Care Nurs Q.

[30] Josey K, Smith M, Kayani A (2018). Hospitals with more-active participation in conducting standardized in-situ mock codes have improved survival after in-hospital cardiopulmonary arrest. Resuscitation.

